# Supplementary material for: The effects of assessing character strengths vs. psychopathology on mood, hope, perceived stigma and cognitive performance in individuals with psychosis
Source: PLoS One. 2023 Aug 10;18(8):e0289872. doi: 10.1371/journal.pone.0289872 (PMC10414607; doi:10.1371/journal.pone.0289872)
Supplement: Attachment — Submitted filename: Response to Reviewers.docx [file pone.0289872.s001.docx]

REVISION - The Effects of Assessing Character Strengths vs. Psychopathology on Mood, Hope, Perceived Stigma and Cognitive Performance in Individuals with Psychosis – Response Letter

Dear Editor, dear Reviewers,

thank you very much for taking the time to thoroughly review our manuscript. We found your comments to be very helpful and believe that implementing them has truly enhanced the paper. In the following, we will go through point by point and explain the changes we have made.

***Editor***

***Based upon these reviews and also my own reading of your manuscript, I think that the results of your study needs to be discussed in a more substantial way. Specifically, you found negative associations between character strenghts (and virtues) and negative symptoms (also with some depressive symptoms). I considere that these results are important for the understanding of psychological features in individuals with psychosis, not only in descriptive terms but also are important to intervention. As a consequence, I invite you to help the readers to better understand these relations.***

To address this point, we have elaborated further in the discussion part on how the possible relationship between character strengths and negative symptoms in individuals with psychosis could look like and what therapeutic implications could arise from it. For this purpose, we present a cognitive model that describes how dysfunctional beliefs, self-image, motivational factors, negative symptoms, and functionality are interconnected, with particular reference to a study by Beck et al (2018) that proposed that the role of a neurological neurocognitive deficit for the functionality of individuals with psychosis could be overestimated, and that dysfunctional beliefs and motivational and attitudinal factors also have an important influence on neurocognitive performance and the level of functioning. Given these relations, it appears to be crucial for the treatment of schizophrenia, especially for negative symptoms and poor level of functioning, to inactivate dysfunctional beliefs and enhance positive attitudes and views of the self. Identifying and fostering character strengths in individuals with psychosis may constitute a fruitful approach for that.

We added the following part to the discussion:

Correlations in the present study were most prominent with negative symptoms, hence the higher negative symptoms the less strongly various strengths are endorsed. This is consistent with cognitive theories of negative symptoms that state that a broadly negative self-image and negative (e.g., defeatist performance-) beliefs could contribute to amotivation, anhedonia, and avolition [23].

Addressing negative symptoms is highly important given the role for long-term functioning. This is supported by the aforementioned findings of Beck et al. [7], which suggest that motivational and attitudinal factors that are severely impaired in individuals with negative symptoms (e.g. amotivation, low effort, social withdrawal) have a significant impact on cognitive performance and the level of functioning. These motivational and attitudinal factors are in turn likely based upon dysfunctional beliefs, such as defeatist performance beliefs. Given these cognitive models of negative symptoms and functionality, it appears to be of great importance for the treatment of schizophrenia to inactivate and weaken dysfunctional beliefs and enhance positive attitudes and views of the self. Based on this principle, Aaron T. Beck and colleagues have developed a therapeutic approach, namely recovery-oriented cognitive therapy (CT-R) [25], which has shown to be particularly promising for problems that are difficult to treat with medication, such as negative symptoms and poor functional outcomes. This is supported by the results of a randomized controlled trial conducted by Grant et al. [26], in which low-functioning patients with schizophrenia were randomly assigned to either receive CT-R or treatment as usual (TAU). The study found that the individuals who received CT-R showed significantly stronger improvements in global functioning and significantly stronger reductions in negative and positive symptoms compared to the TAU group. Moreover, a six-month follow-up assessment showed that the significant benefits of recovery-oriented cognitive therapy over standard treatment were maintained with comparable effect sizes [27]. The negative correlations found in this study between negative symptoms and character strengths could indicate that identifying and promoting character strengths in individuals with psychotic disorders may represent a fruitful therapeutic approach in the sense of recovery-oriented cognitive therapy, especially concerning negative symptoms. (p.14 lines 315-337)

***Reviewer 1***

***1. There are a few linguistic corrections that should be made (e.g. “acceptance of focusing character strengths”, p. 9; “developed by Timo Gnambs for in Unipark”, p. 11)***

We went over the whole manuscript once again and corrected the linguistic errors. Thank you for paying close attention and pointing them out.

***2. In the study, the 120-version of the VIA-IS was used. The full version comprises 240 items, and it should hence be mentioned that the short version was used, and the appropriate citation and reference should be added.***

We added this information with the appropriate citation and reference:

Character strengths were measured with the 120 items short version of the Values in Action Inventory of Strengths [5] in a validated German version [17]. In total, 24 character strengths are assessed with 5 items per strength. (p.7 lines 170-172)

5. Peterson, C., & Seligman, M. E. P. (2004). Character strengths and virtues: A handbook and classification. New York: Oxford University Press and Washington, DC: American Psychological Association. (p.17 lines 388-390)

***3. Please report the reliabilities of each scale of the employed measures across the full sample. These could be summarised with range and median for the VIA-IS scales given the large number of scales.***

The Cronbach’s alpha coefficients of the scales of the employed measures across the sample sample of this study read as follows:

- State Optimism Measure (Millstein et al., 2019): α=.97
- Brief Symptom Inventory (Derogatis & Melisaratos, 1983): α=.94
- Perceived Stigma (Alonso et al, 2008)(state-adapted short form, see manuscript): α=.93
- Positive and Negative Affect Schedule (Crawford & Henry, 2004): α_positive_=.86; α_negative_=.76 (two subscales: positive affect and negative affect)
- ZUF-8 (patient satisfaction form; Kriz et al, 2008)(customized version, see manuscript): α=.93
- Community Assessment of Psychic Experiences (Konings et al, 2006): α_positive_=.90; α_negative_=.88; α_depressive_=.81 (three subscales: positive symptoms, negative symptoms and depressive symptoms)
- University of Rhode Island Change Assessment Scale (Hasler et al, 2003): α=.79
- Values in Action Inventory of Strengths 120 item short version (Peterson & Seligman, 2004): Median of α for VIA-scales=.72; range of α for VIA-scales: .33 - .90
- 4-items psychosis lie scale (Moritz et al, 2013): α= .62

We have added the Cronbach's alpha coefficients for the scales, respectively, behind their descriptions in the measurements section of the manuscript. Additionally, we have included the following sentence in the discussion section:

The reported reliabilities of the scales, which refer to the sample of this study, were generally good (α >.80), however three of the VIA-IS scales for the character strengths showed unsatisfactory reliability of a Cronbach’s α<.60 (i.e., *fairness*, α=.59; *kindness*, α=.51; *prudenc*e, α=.32), which should be taken into account when interpreting results concerning these three subscales. (p.16 lines 365-369)

***4. In Table 2, it is not explained why certain values are in bold and what the asterisks indicate. Please add this information in the Note. Furthermore, as the correlations were across a small sample, Pearson correlations could be over- or underestimated due to outliers, even if each variable is approximately normal distributed. Hence, I would rather recommend conducting Spearman rank correlations throughout to avoid these biases***.

We followed your recommendation and conducted Spearman rank correlations throughout to check associations between psychotic symptom domains and character strengths. This resulted in the following table: (p.11, 12 lines 279-282)

**Table 2. Spearman-rho correlations of character strengths with psychotic symptoms and depression CAPE mean frequency scores (*N* = 39).**

| *Domain* | *Character*  *strength* | *Positive*  *Symptoms* | *Negative*  *Symptoms* | *Depression* |
| --- | --- | --- | --- | --- |
| **Wisdom & knowledge** | Creativity | .092 | *-.308^†^* | -.055 |
|  | Curiosity | .149 | **-.555^***^** | -.201 |
|  | Judgment | -.114 | .230 | .219 |
|  | Love of learning | .170 | *-.289^†^* | .066 |
|  | Perspective | .037 | -.129 | -.005 |
| **Courage** | Bravery | .154 | **-.370^*^** | -.076 |
|  | Persistence | .083 | **-.343^*^** | -.082 |
|  | Authenticity | .171 | -.042 | .119 |
|  | Enthusiasm/ zest | .087 | **-.755^***^** | **-.418^**^** |
| **Humanity** | Love/ affiliation | .092 | -.216 | -.141 |
|  | Kindness | .101 | -.164 | .144 |
|  | Social intelligence | .151 | **-.355^*^** | .007 |
| **Justice** | Teamwork | .048 | **-.438^**^** | *-.286^†^* |
|  | Fairness | **.430^**^** | -.065 | .203 |
|  | Leadership | **.421^**^** | -.128 | .176 |
| **Temperance** | Forgiveness | .024 | -.242 | .011 |
|  | Humility | .005 | -.211 | -.080 |
|  | Prudence | .151 | -.041 | .070 |
|  | Self-control | -.112 | **-.549^***^** | **-.507^**^** |
| **Transcendence** | Appreciation | .106 | -.165 | **.324^*^** |
|  | Gratitude | -.116 | **-.622^***^** | **-.332^*^** |
|  | Hope/ optimism | -.195 | **-.541^***^** | **-.553^***^** |
|  | Humor | .113 | -.211 | .114 |
|  | Spirituality | -.190 | **-.403^*^** | -.183 |

**Note.** *^†^ p < .10, * p <.05, ** p < .01, *** p <.001*

Compared to our previous analysis where we used Pearson correlation for variables with normal distribution, slight changes have emerged. Specifically, we now observe positive correlations for two instead of three character strengths with positive symptoms, negative correlations for 10 instead of 12 character strengths with negative symptoms, and for depression, we now observe negative correlations with 4 instead of 3 character strengths, as well as one positive correlation with a character strength. Correlations are still most prominent with negative symptoms.

As you can see, we have added in the Note what the asterisks indicate. Bold values indicate significant correlations.

In addition to the changes in this table, we made the following changes to the manuscript:

Ten of the 24 character strengths were significantly and negatively correlated with negative symptoms (see Table 2). Positive correlations with positive symptoms were identified for two character strengths (fairness, leadership). For depression there were four negative correlations and one positive correlation with character strengths (for details see Table 2). (p.11 lines 274-277)

***Reviewer 2***

***The introduction is too short and could benefit to cite some other works on effects of attitudinal or motivational factors on neurocognitive performance. For example: Beck, A.T., Himelstein, R., Bredemeier, K., Silverstein, S.M., Grant, P. (2018). What accounts for poor functioning in people with schizophrenia: A re-evaluation of the contributions of neurocognitive v. attitudinal and motivational factors. Psychol Med. 48(16):2776-85.***

We appreciate your reference to the paper by Beck and colleagues. The insights provided therein complement our introduction wonderfully, aiding the reader in understanding the role that non-neurocognitive factors play in schizophrenia, and why positive psychological interventions might be relevant in this context. Additionally, there was a significant congruence between the findings presented in this paper and another paper cited in our introduction (Moritz et al, 2021). What we made of these insights for the paper is the following:

On the other hand, the impact of negative views upon the self, or self- / social stigma, can have detrimental effects on different levels, including mental health and functioning of an individual or certain, stigmatized groups. Recent work implies that the role of (biologically determined) neurocognitive deficits in the level of functioning in individuals with schizophrenia may have been overemphasized and overestimated. It has been shown that neurocognitive performance is significantly influenced by attitudinal and motivational factors such as dysfunctional beliefs, avolition, and negative emotions. Beck et al. [7] propose a cognitive model based on dysfunctional beliefs that provides a comprehensive approach to explain poor performance in laboratory tasks and poor level of functioning among individuals with schizophrenia, challenging the assumption that there is a neurologically-based neurocognitive deficit in schizophrenia that is independent of other psychological processes. The core component of the therapy derived from this model, labeled as recovery-oriented cognitive therapy (CT-R), is to strengthen positive beliefs and weaken negative beliefs. Moritz et al. [8] come to the same conclusion that the neurocognitive deficits in schizophrenia might have been overestimated, among other factors partially due to motivational effects, stereotypes or even stigma (i.e., pre-supposing cognitive deficits), or defeatists beliefs, and that taking these factors into account opens new opportunities for treatment. (p.3 lines 71-87)

***Furthermore, recent development of Cognitive Therapy [Beck, A.T., Grant, P.M., Inverso, E., Brinen, A.P., & Perivoliotis, D. (2020). Recovery oriented cognitive therapy for serious mental health conditions. Guilford Press; Grant, P.M., Bredemeier, K., & Beck, A.T. (2017). Six-month follow-up of recovery-oriented cognitive therapy for low functioning individuals with schizophrenia. Psychiatric Services, 68, 997–1002] could be cited and discussed in the discussion section.***

The book ‘*Recovery oriented cognitive therapy for serious mental health conditions’* by Beck et al (2020) is very helpful in gaining a comprehensive understanding of the cognitive model underlying recovery-oriented cognitive therapy and the therapeutic principles upon which it is based. We hope that we have been able to convey its essential aspects in our discussion and that the connection to addressing character strengths in individuals with psychosis has become clear. The study by Grant et al (2017), that you cited, demonstrates that CT-R is evidence-based and a promising therapeutic approach for the treatment of negative symptoms, positive symptoms, and poor functionality. We have also incorporated this study into our discussion, the changes made read as follows:

Addressing negative symptoms is highly important given the role for long-term functioning. This is supported by the aforementioned findings of Beck et al. [7], which suggest that motivational and attitudinal factors that are severely impaired in individuals with negative symptoms (e.g. amotivation, low effort, social withdrawal) have a significant impact on cognitive performance and the level of functioning. These motivational and attitudinal factors are in turn likely based upon dysfunctional beliefs, such as defeatist performance beliefs. Given these cognitive models of negative symptoms and functionality, it appears to be of great importance for the treatment of schizophrenia to inactivate and weaken dysfunctional beliefs and enhance positive attitudes and views of the self. Based on this principle, Aaron T. Beck and colleagues have developed a therapeutic approach, namely recovery-oriented cognitive therapy (CT-R) [25], which has been shown to be particularly promising for problems that are difficult to treat with medication, such as negative symptoms and poor functional outcomes. This is supported by the results of a randomized controlled trial conducted by Grant et al. [26], in which low-functioning patients with schizophrenia were randomly assigned to either receive CT-R or treatment as usual (TAU). The study found that the individuals who received CT-R showed significantly stronger improvements in global functioning and significantly stronger reductions in negative and positive symptoms compared to the TAU group. Moreover, a six-month follow-up assessment showed that the significant benefits of recovery-oriented cognitive therapy over standard treatment were maintained with comparable effect sizes [27]. The negative correlations found in this study between negative symptoms and character strengths could indicate that identifying and promoting character strengths in individuals with psychotic disorders may represent a fruitful therapeutic approach in the sense of recovery-oriented cognitive therapy, especially concerning negative symptoms. (p.14 lines 315-337)

***Moreover, the rationale and aims of the study should be present more clearly.***

After re-reading the introduction, we found this point to be very accurate and have accordingly added the following paragraph to clarify the rationale and objectives of our study:

Given the findings on the role of dysfunctional beliefs in neurocognitive performance and level of functioning in schizophrenia, the potential beneficial effects of directing attention towards positive self-attributes, and the currently limited research on addressing character strengths in psychosis, we conducted this study to contribute to the discussion on whether focusing on character strengths could be an important contribution to alleviate the stereotypical, deficit-oriented view on individuals with schizophrenia and also their view of themselves. In the long run, working with character strengths might then improve symptomatic and functional outcomes. To begin, we took one step back and investigated whether merely measuring psychopathology vs. character strengths may already have an effect on psychological states. In addition, the present study aimed at exploring correlations between character strengths and psychotic symptoms to potentially identify specific symptom domains for which addressing character strengths could be particularly fruitful in treatment. Finally, the general acceptance of focusing on character strengths was investigated. (p.4 lines 100-112)

***The methods section is well presented. However, the description of the procedure seems too wordy and difficult to follow. It could be described more clearly.***

We have shortened and simplified this part of the manuscript, now it reads as follows:

## Procedure

The study used various recruitment methods, including flyers and social media, as well as a mailing list of former study participants from the Medical Center Hamburg-Eppendorf (UKE). Inclusion criteria were age > 18 years, self-reported formal diagnosis of a psychotic disorder, sufficient emotional stability, and being able and willing to give informed consent after reading the study information. The study was conducted online and designed as a randomized-controlled within-subject cross-over study.

Once participants provided informed consent, they were assessed for cognitive function using the Trail Making Test A and B (TMT A&B), as well as psychological states (mood, optimism, therapy motivation, and perceived stigma), which served as the baseline measures.

Participants were then randomly assigned to either a psychopathology assessment block or a character strengths assessment block. After completing the assigned assessment block, participants underwent another round of assessment of the TMT A&B and psychological states. They then completed the respective other assessment block (psychopathology or character strengths), followed by a final assessment of the TMT A&B and psychological states.

After that, all participants received a brief character strength intervention, which consisted of personalized feedback on their top-two character strengths, as well as an instruction sheet with easy-to-implement exercises to make use of their character strengths in everyday life.

At the end of the study, socio-demographic information, including current or past diagnoses and therapy status, were assessed. To distinguish potential ‘simulators’ from real patients in this online study, a 4-item psychosis lie scale (cutoff: 8 points); [11] was used (α=.62), which was integrated within the CAPE (see Psychopathology subsection for details), whereby the respective items reflect clichés about psychosis, but are, in fact, rather uncommon (e.g., alien abduction). Scores beyond cutoff speak for simulation of psychosis. This study, including all conducted analyses, was conducted with the formal approval of the ethics committee of the University Medical Center Hamburg-Eppendorf (LPEK-0166, approval date 06.07.2020). (p.4, 5 lines 114-132)

***In the results section (Table 1) TMT, negative affect and stigma appears to be significantly different between baseline and the two assessment blocks. What does it mean? Authors should describe it and try to explain these differences.***

We actually missed this in the original version of the paper. Thanks for your observation of these differences. We have now reported these differences in the results section and offered possible explanations in the discussion section:

There were no differences in the psychological states ratings or cognitive performance between the pathology vs. character strengths assessments. Time effects were observed, with the variables TMT, negative affect and stigma decreasing in both conditions compared to baseline. For statistical details, see Table 1. (p.10 lines 255-257)

However, there were small-sized time effects that emerged in the form of decreased negative affect and perceived stigma, as well as improved cognitive performance (measured by the TMT), in comparison to baseline, for both the pathology focus and the character strengths focus. The reduced completion times for the TMT tasks may potentially be attributed to a learning effect. The reduction in negative affect and perceived stigma in both conditions could be interpreted as the result of the comprehensive assessment of symptoms and character strengths within the scope of this study, which may have led the participants to feel more perceived and less discriminated. In addition or alternatively, anticipatory anxiety or stress at beginning of the study may have simply decreased over time (regression to the mean). As suggested by some studies, the phenomenon of initial elevation bias, whereby higher (especially negative) states are reported in initial assessments compared to later assessments, appears to be not uncommon in research utilizing subjective reports [23].

(p.13 lines 294-305)

***Although the authors describe the main limitations of the study at the end of their manuscript, sample size and heterogeneity, sample recruitment (self-reported rather than formal diagnosis), and the decision not to measure the potential effect of the strengths character feedback intervention should be addressed more extensively.***

We have now discussed the limitations for this study much more extensively and hope that we have been able to adequately address each point that you have raised:

Limitations of the present study are, for one, the sampling approach. Some individuals reported a first episode (20.5%), most reported multiple episodes (69.2%), and some only past episodes (10.3%). The effects of the intervention could differ by the degree of chronification and this should be further investigated in future studies with larger samples, whereby moderation effects could be examined. Another point of criticism is the reliance on an online format and hence self-reported diagnoses, lacking a differentiated formal diagnosis (e.g., using the SCID-5). However, applying a lie scale, and descriptively comparing symptom scores of the CAPE in the current sample to other samples, suggested plausible expressions of psychotic symptoms in the present sample. As a matter of fact, the study was initially planned to be conducted face-to-face which would have allowed for a diagnostic assessment, but was then conducted online due to strict COVID-19 policies in Germany during the planned recruitment period. A face-to-face format could have furthermore elicited stronger effects of the manipulations, since a social component of the experimenter conducting the assessments and, perhaps, also giving feedback, could have had a much stronger impact upon the participants’ psychological states and performance. Another reason for the manipulation not being successful might have been that individuals could have self-criticized internally for lacking the strength at question, albeit the general improvement over time in negative affect, perceived stigma, and cognitive performance somewhat contradicts this notion. Instead of relying on longer blocks of assessment, and to separate the manipulation from the rest of the study, it would have been beneficial to work with feedback on strengths vs. feedback on deficits or pathology. In addition, it is possible that attempting a psychological state change induction twice and in opposite hypothesized directions during one experiment (within sample) may have been too ambitious – hence replication studies may use longitudinal or between-subject designs. Finally, the focus of the study was on the effect of assessing strengths vs. deficits, and not the effect of character strength feedback per se. However, evaluating the effect of the strength feedback on psychological states might have been insightful beyond the assessment of overall perceived relevance of the topic and subjective benefit, and this should be implemented in future studies. The reported reliabilities of the scales, which refer to the sample of this study, were generally good (α >.80), however three of the VIA-IS scales for the character strengths showed unsatisfactory reliability of a Cronbach’s α<.60 (i.e., *fairness*, α=.59; *kindness*, α=.51; *prudenc*e, α=.32), which should be taken into account when interpreting results concerning these three subscales.

(p. 14, 15, 16 lines 338-369)

In addition to the previously described changes to the manuscript, we made two other minor adjustments:

- We have added the information that in the context of our correlation analyses between character strengths and psychotic symptom domains, we used the mean of the frequency dimension of the CAPE subscales.
- The central outcome parameter for the cognitive test in this study was actually TMT-B minus TMT-A and not TMT-B divided by TMT-A as we had incorrectly stated in the first version of the paper. However, a re-analysis with both methods did not reveal any differences in significant effects.

**References**

1. Bonsack C. The roots of the recovery movement in psychiatry. Psychosis. 2012; 4: 87–88. doi:10.1080/17522439.2012.643597.
2. Davidson L. The Recovery Movement: Implications For Mental Health Care And Enabling People To Participate Fully In Life. Health Affairs. 2016; 35: 1091–1097. doi: 10.1377/hlthaff.2016.0153.
3. Jeste DV, Palmer BW, Rettew DC, Boardman S. Positive psychiatry: Its time has come. The Journal of Clinical Psychiatry. 2015; 76: 675–683. doi: 10.4088/JCP.14nr09599.
4. Csikszentmihalyi M, Csikszentmihalyi IS. *A Life Worth Living: Contributions to Positive Psychology*. Oxford University Press; 2006.
5. Peterson C, Seligman MEP. Character strengths and virtues: A handbook and classification. New York: Oxford University Press and Washington, DC: American Psychological Association; 2004.
6. Schutte NS, Malouff JM. The Impact of Signature Character Strengths Interventions: A Meta-analysis. Journal of Happiness Studies. 2019; *20*: 1179–1196. doi: 10.1007/s10902-018-9990-2.
7. Beck AT, Himelstein R, Bredemeier K, Silverstein SM, Grant P. What accounts for poor functioning in people with schizophrenia: A re-evaluation of the contributions of neurocognitive v. attitudinal and motivational factors. Psychol Med. 2018; 48: 2776-85.
8. Moritz S, Silverstein SM, Beblo T, Özaslan Z, Zink M, Gallinat J. Much of the Neurocognitive Impairment in Schizophrenia is Due to Factors Other Than Schizophrenia Itself: Implications for Research and Treatment. Schizophrenia Bulletin Open. 2021; 2: sgaa034. doi: 10.1093/schizbullopen/sgaa034.
9. Browne J, Estroff SE, Ludwig K, Merritt C, Meyer-Kalos P, Mueser KT, et al. Character strengths of individuals with first episode psychosis in Individual Resiliency Training. Schizophrenia Research. 2018; 195:448–454. doi: 10.1016/j.schres.2017.09.036.
10. Sims A, Barker C, Price C, Fornells-Ambrojo M. Psychological impact of identifying character strengths in people with psychosis. *Psychosis*. 2015; *7*: 179–182. doi: 10.1080/17522439.2014.925485.
11. Moritz S, Van Quaquebeke N, Lincoln TM, Köther U, Andreou C. Can We Trust the Internet to Measure Psychotic Symptoms? Schizophrenia Research and Treatment. 2013; 2013: e457010. doi: 10.1155/2013/457010.
12. Reitan RM. TMT, Trail Making Test A & B. South Tucson, AR: Reitan Neuropsychology Laboratory; 1992.
13. Crawford JR, Henry JD. The Positive and Negative Affect Schedule (PANAS): Construct validity, measurement properties and normative data in a large non-clinical sample. British Journal of Clinical Psychology. 2004; 43: 245–265. doi: 10.1348/0144665031752934.
14. Hasler G, Klaghofer R, Buddeberg C. The university of rhode island change assessment scale (URICA). Psychotherapie, Psychosomatik, Medizinische Psychologie. 2003; 53: 406–411.
15. Millstein R, Chung WJ, Hoeppner B, Boehm J, Legler S, Mastromauro C, et al. Development of the State Optimism Measure. General Hospital Psychiatry. 2019; *58*: 83-93.doi: 10.1016/j.genhosppsych.2019.04.002.
16. Alonso J, Buron A, Bruffaerts R, He Y, Posada‐Villa J, Lepine JP, et al. Association of perceived stigma and mood and anxiety disorders: Results from the World Mental Health Surveys. Acta Psychiatrica Scandinavica. 2008;118: 305–314. doi:10.1111/j.1600-0447.2008.01241.
17. Ruch W, Proyer RT, Harzer C, Park N, Peterson C, Seligman MEP. Values in Action Inventory of Strengths (VIA-IS): Adaptation and validation of the German version and the development of a peer-rating form. Journal of Individual Differences. 2010; 31: 138–149. doi:10.1027/1614-0001/a000022.
18. McGrath RE. Scale- and Item-Level Factor Analyses of the VIA Inventory of Strengths. Assessment. 2014**;** 21: 4–14. doi: 10.1177/1073191112450612.
19. Derogatis LR., Melisaratos N. The Brief Symptom Inventory: An introductory report. Psychological Medicine. 1983; 13: 595–605.
20. Konings M, Bak M, Hanssen M, Van Os J, Krabbendam L. Validity and reliability of the CAPE: A self-report instrument for the measurement of psychotic experiences in the general population. Acta Psychiatrica Scandinavica. 2006; 114:55–61. doi: 10.1111/j.1600-0447.2005.00741.
21. Kriz D, Nübling R, Steffanowski A, Wittmann WW, Schmidt J. Patientenzufriedenheit in der stationären Rehabilitation: Psychometrische Reanalyse des ZUF-8 auf der Basis multizentrischer Stichproben verschiedener Indikation. Zeitschrift Für Medizinische Psychologie. 2008; 17: 67–79.
22. Jaya ES, van Amelsvoort T, Bartels-Velthuis, AA, Bruggeman R, Cahn W, de Haan L, et al. The Community Assessment of Psychic Experiences: Optimal cut-off scores for detecting individuals with a psychotic disorder. International Journal of Methods in Psychiatric Research. 2021; 30: e1893. doi: 10.1002/mpr.1893.
23. Arslan RC, Reitz AK, Driebe JC, Gerlach TM, Penke L. Routinely randomize potential sources of measurement reactivity to estimate and adjust for biases in subjective reports. Psychological Methods. 2021; 26: 175-185. doi: 10.1037/met0000294
24. Rector NA, Beck AT, Stolar N. The negative symptoms of schizophrenia: A cognitive perspective. Canadian Journal of Psychiatry. Revue Canadienne De Psychiatrie. 2005; 50: 247–257.
25. Beck AT, Grant PM, Inverso E, Brinen AP, Perivoliotis D. Recovery oriented cognitive therapy for serious mental health conditions. Guilford Press; 2020.
26. Grant PM, Huh GA, Perivoliotis D, Stolar NM and Beck AT. Randomized trial to evaluate the efficacy of cognitive therapy for low- functioning patients with schizophrenia. Archives of General Psychiatry. 2012; 69: 121–127.
27. Grant, PM, Bredemeier K, Beck AT. Six-month follow-up of recovery-oriented cognitive therapy for low functioning individuals with schizophrenia. Psychiatric Services. 2017; 68: 997–1002.
