# Supplementary material for: The effects of assessing character strengths vs. psychopathology on mood, hope, perceived stigma and cognitive performance in individuals with psychosis
Source: PLoS One. 2023 Aug 10;18(8):e0289872. doi: 10.1371/journal.pone.0289872 (PMC10414607; doi:10.1371/journal.pone.0289872)
Supplement: Attachment — Submitted filename: Response to Reviewers .docx [file pone.0289872.s002.docx]

REVISION#2 - The effects of assessing character strengths vs. psychopathology on mood, hope, perceived stigma and cognitive performance in individuals with psychosis – Response Letter

Dear Editor, dear Reviewers,

we would like to thank you once again for taking the time to thoroughly review our manuscript and for identifying additional areas for improvement. We will address these below.

***Reviewer 1***

***1. The newly added references for the VIA-IS short version both refer to the original, long versions of the questionnaire (with 240 items). The references for the short versions are Littman-Ovadia (2015) for the English version and Höfer, Hausler, Huber, Strecker, Renn and Höge (2020) for the German version.***

We have now added the appropriate references for the VIA-IS short versions to our manuscript:

Character strengths were measured with the 120 items short version of the Values in Action Inventory of Strengths [17] in a validated German version [18]. (p.7 lines 170-171)

17. Littman-Ovadia H. Brief Report: Short Form of the VIA Inventory of Strengths- Construction and Initial Tests of Reliability and Validity. International Journal of Humanities Social Sciences and Education. 2015; 2: 229-237.
18. Höfer S, Hausler M, Huber A, Strecker C, Renn D, Höge T. Psychometric characteristics of the German values in Action Inventory of Strengths 120-item short form. Applied Research in Quality of Life. 2020; 15: 597-611. doi: 10.1007/s11482-018-9696-y. (p.19 lines 430-436)

***2.*** ***The labelling of the character strengths in Figure 1, Table 2 and the text is inconsistent. It would be helpful to label them consistently throughout the manuscript.***

We followed your advice and labelled the character strengths consistently throughout the manuscript:

The 24 strengths in turn are assigned to 6 superordinate human virtues: *wisdom and knowledge* (strengths: creativity, curiosity, judgment, love of learning, perspective), *courage* (strengths: bravery, perseverance, honesty, zest), *humanity* (strengths: love, kindness, social intelligence), *justice* (strengths: teamwork, fairness, leadership), *temperance* (strengths: forgiveness, humility, prudence, self-regulation) and *transcendence* (strengths: appreciation of beauty and excellence, gratitude, hope, humor, spirituality), although the factorial structure of the questionnaire has been challenged in later factor analyses [19]. (p.7 lines 172-179)

Kindness, honesty and judgment were the three descriptively most highly endorsed character strengths; the three least endorsed character strengths were self-regulation, spirituality, and humility. For a full average profile, see Fig 1. (p.11 lines 265-267)

Positive correlations with positive symptoms were identified for two character strengths (fairness, leadership). (p.11 lines 274-275)

Self-regulation descriptively is the least, kindness the most strongly endorsed strength in the current sample. Kindness was also among the most endorsed strengths in a sample with first episode psychosis, as were honesty and fairness [9]. (p.13 lines 306-308)

The reported reliabilities of the scales, which refer to the sample of this study, were generally good (α >.80), however three of the VIA-IS scales for the character strengths showed unsatisfactory reliability of a Cronbach’s α<.60 (i.e., *fairness*, α=.59; *kindness*, α=.51; *prudenc*e, α=.32), which should be taken into account when interpreting results concerning these three subscales. (p.16 lines 366-367)

**Table 2. Spearman-rho correlations of character strengths with psychotic symptoms and depression CAPE mean frequency scores (*N* = 39).**

| *Domain* | *Character*  *strength* | *Positive*  *symptoms* | *Negative*  *symptoms* | *Depression* |
| --- | --- | --- | --- | --- |
| **Wisdom & Knowledge** | Creativity | .092 | *-.308^†^* | -.055 |
|  | Curiosity | .149 | **-.555^***^** | -.201 |
|  | Judgment | -.114 | .230 | .219 |
|  | Love of Learning | .170 | *-.289^†^* | .066 |
|  | Perspective | .037 | -.129 | -.005 |
| **Courage** | Bravery | .154 | **-.370^*^** | -.076 |
|  | Perseverance | .083 | **-.343^*^** | -.082 |
|  | Honesty | .171 | -.042 | .119 |
|  | Zest | .087 | **-.755^***^** | **-.418^**^** |
| **Humanity** | Love | .092 | -.216 | -.141 |
|  | Kindness | .101 | -.164 | .144 |
|  | Social Intelligence | .151 | **-.355^*^** | .007 |
| **Justice** | Teamwork | .048 | **-.438^**^** | *-.286^†^* |
|  | Fairness | **.430^**^** | -.065 | .203 |
|  | Leadership | **.421^**^** | -.128 | .176 |
| **Temperance** | Forgiveness | .024 | -.242 | .011 |
|  | Humility | .005 | -.211 | -.080 |
|  | Prudence | .151 | -.041 | .070 |
|  | Self-Regulation | -.112 | **-.549^***^** | **-.507^**^** |
| **Transcendence** | Appreciation of Beauty and Excellence | .106 | -.165 | **.324^*^** |
|  | Gratitude | -.116 | **-.622^***^** | **-.332^*^** |
|  | Hope | -.195 | **-.541^***^** | **-.553^***^** |
|  | Humor | .113 | -.211 | .114 |
|  | Spirituality | -.190 | **-.403^*^** | -.183 |

**Note.** ^†^ *p* < .10, * *p* <.05, ** *p* < .01, *** *p* <.001 (p.11, 12 lines 278-281)

(See figure file Fig1.tif)

***Reviewer 2: (No Comments to the Author)***
